# Flavoprotein miniSOG Cytotoxisity Can Be Induced By Bioluminescence Resonance Energy Transfer

**Published:** 2016

**Authors:** E.I. Shramova, G.M. Proshkina, S.P. Chumakov, Yu.M. Khodarovich, S.M. Deyev

**Affiliations:** Shemyakin/Ovchinnikov Institute of Bioorganic Chemistry, Russian Academy of Sciences, Miklukho-Maklaya Str., 16/10, 117997, Moscow, Russia; National Research Tomsk Polytechnic University, Lenina av., 30, 634050, Tomsk, Russia

**Keywords:** bioluminescence resonance energy transfer, NanoLuc luciferase, flavoprotein miniSOG, photodynamic therapy

## Abstract

In this study, we investigated the possibility of phototoxic flavoprotein miniSOG (photosensitizer) excitation in cancer cells by bioluminescence occurring when luciferase NanoLuc oxidizes its substrate, furimazine. We have shown that the phototoxic flavoprotein miniSOG expressed in eukaryotic cells in fusion with NanoLuc luciferase is activated in the presence of its substrate, furimazine. Upon such condition, miniSOG possesses photoinduced cytotoxicity and causes a 48% cell death level in a stably transfected cell line.

## INTRODUCTION

The properties of light as a therapeutic agent have been used by mankind for over 3,000 years [1]. The starting point of our modern approach to the study of the photosensitivity phenomenon is considered to be the work of Oscar Raab published in 1900 [2]. Raab revealed that the combination of light with certain chemicals induces the death of living cells: the acridine orange dye causes the death of ciliates on a sunny day, but not on a cloudy day [2]. Modern photodynamic therapy (PDT) appeared with the discovery of this fact.

The modern form of the photodynamic therapy is a three-component system consisting of a photosensitizer, a light of a certain wavelength, and molecular oxygen. These three key elements, each individually non-toxic, produce reactive oxygen species (ROS) when combined and, thus, induce oxygen-mediated cell death.

PDT is a promising method for the treatment of human malignant tumors, because it allows for selective and local action on the tumor.

Because photodynamic therapy requires an external light source, the method is applied only in the treatment of skin and retina tumors in clinical practice, as well as the epithelial surfaces of organs accessible to catheters and endoscopes. For example, PDT is now approved for the treatment of head and neck carcinomas [3], lung cancer [4], the upper digestive tract [5], and malignancies [6].

The main obstacle of photodynamic therapy is related to a loss of the optical activity (intensity) of the exciting light as a result of refraction, reflection, absorption and dispersion of light quanta in biological tissues. Due to the ability of tissues to absorb and disperse light, the penetrating power of visible light in tissues does not exceed 10 mm. Moreover, light absorption is determined by the biological chromophores of the tissue: almost all proteins are target chromophores in the ultraviolet region of the spectrum, oxyhemoglobin, deoxyhemoglobin, and melanin absorb light with a wavelength of 400 to 600 nm, while water absorbs light with a wavelength of 1,200 to 2,000 nm. Thus, the “optical window” of biological tissues for PDT is in the range of 650–1,200 nm [7].

With the onset of metastasis, it becomes difficult or impossible to deliver light directly to all tumor growth foci. In the case if internal light sources are developed, the light can be delivered to any body area and to any depth, which can significantly expand the scope of photodynamic therapy application [8].

**Fig. 1:**
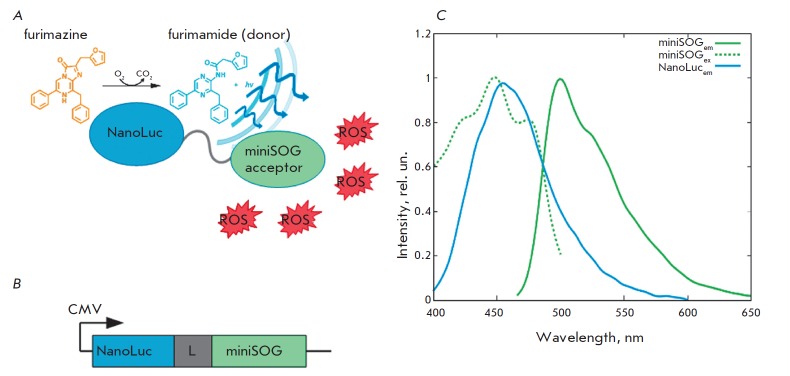
Bioluminescence system based on luciferase, furimazine, and miniSOG. *A *– Schematic illustration of the BRET system for PDT. *B *– Gene construct encoding NanoLuc, peptide linker GGGGS and cytotoxic module miniSOG within one reading frame. *C *– Normalized emission spectrum of furimamide (NanoLuc_em_) and normalized excitation (miniSOG_ex_) and emission (miniSOG_em_) spectra of miniSOG.

The phenomenon of bioluminescence resonance energy transfer (BRET) is widely used in modern molecular and cell biology for *in vivo *and *in vitro *study of intracellular processes, as well as for bioimaging [9-11]. BRET is based on Förster resonance energy transfer between two chromophores, where a luciferase substrate serves as the donor, which is oxidized in the presence of oxygen and emits photons in the visible spectrum, while a fluorescent protein acts as the acceptor (*Fig. 1A*).

With the advances in gene therapy approaches, the gene encoding luciferase can be selectively expressed in tumor cells using tumor-specific promoters [12, 13] or selectively delivered to tumor cells using such vehicles as pseudotyped viral vectors [14] targeting polyethyleneimine complexes, etc. [15]. Thus, the use of bioluminescence as an intracellular source of light for the excitation of the photosensitizer in a cancer cell may serve as a solution to the problem of light delivery to the deep regions of tissues.

The applicability of the phenomenon in therapy was first demonstrated in 1994 [16]: photosensitizer hypericin excited by luciferin bioluminescence led to *in vitro *inactivation of the equine infectious anemia virus.

However, the use of BRET in the photodynamic therapy of cancer was demonstrated only in 2003 [17]. The photosensitizer bengal red located in the cytosol in the presence of luciferin caused the death of 90% of a population of NIH 3T3 mouse fibroblasts stably expressing the luciferase gene.

According to [18], the luminescent molecule luminol can also be used as an intracellular light source for the excitation of the photosensitizer. The viability of HeLa cells treated with luminol in the presence of the photosensitizer was less than 10%. Anti-tumor therapy *in vivo *led to a 55% decrease in tumor growth in mice of the experimental group compared to the control group. In addition, luminol and the photosensitizer were injected directly into the tumors of the experimental animals.

The possibility of using BRET**-**mediated photodynamic therapy of deep tissue tumors and metastases was demonstrated in a mouse model in 2015 [19]. In that work, quantum dots coated with luciferase were used as an intracellular source for photodynamic therapy. The quantum dots excited the photosensitizer chlorin e6 in the presence of a luciferase substrate, leading to a regression of the primary tumor focus and metastases in the lymph nodes.

We should note that chemical photosensitizers administered intratumorally or systemically to the body were used in all of the mentioned papers.

Targeted genetically encoded protein photosensitizers with high cytotoxic activity against HER2-positive breast adenocarcinoma cells *in vitro *were previously obtained in our laboratory on the basis of the phototoxic flavoprotein miniSOG [20-23]. MiniSOG excitation occurs in the blue region of the spectrum (λ_max_ = 448 nm) [24], which imposes some restrictions on the use of these photosensitizers *in vivo*.

In order to solve the problem of blue light delivery *in vivo*, we propose a system where the miniSOG photosensitizer is excited during luciferase NanoLuc (Promega) oxidation of the substrate (furimazine). We have shown that NanoLuc luciferase expressed in eukaryotic cells as part of the genetic construct with miniSOG causes the excitation of phototoxic flavoprotein in the presence of the furimazine substrate. Moreover, miniSOG exhibits photoinduced cytotoxicity and causes the death of 48% of the transfected cells.

## EXPERIMENTAL SECTION

**Eukaryotic cell cultures**

Human breast adenocarcinoma SK-BR-3 cells were used in this study. The cells were grown in a McCoy’s 5A (HyClone, Belgium) or RPMI 1640 medium without phenol red (Gibco, Germany) containing 10% fetal bovine serum (HyClone, Belgium) and antibiotics (50 U/ml penicillin, 50 μg/ml streptomycin, “PanEco”, Russia) at 37°C and 5% CO_2_ in high humidity. For cultivation of the cells expressing miniSOG, riboflavin (“Pharmstandart-UfaVita”, Russia) was added to the medium as a source of the FMN cofactor to a final concentration of 150 μM.

**NanoLuc–miniSOG construction**

The coding sequence of the photoactivatable cytotoxic miniSOG protein gene was cloned into pNL1.1.CMV (Promega) plasmid containing the NanoLuc luciferase gene under the control of the CMV promoter using standard techniques of genetic engineering. The coding sequence of *miniSOG *was amplified from the pDARP-miniSOG plasmid [22] using the specific primers oGP13 nucleases AvaI and XbaI and cloned into a pNL1.1.CMV vector digested with the same restriction enzymes. As a result, a pNanoLuc-miniSOG plasmid was obtained containing *NanoLuc*- and *miniSOG*-encoding sequences within the same reading frame connected by a linker region under the control of the constitutive promoter CMV. The accuracy of the construct was confirmed by sequencing. The scheme of the genetic construct is presented in *Fig. 1B*.

**pNanoLuc-miniSOG-puro plasmid construction**

To obtain cell lines that stably expressed the *NanoLuc*-*mSOG *fusion gene, the puromycin resistance gene was introduced into the NanoLuc-miniSOG plasmid. This gene, including the NP promoter of the human *p53 *gene and polyadenylation signal, was amplified from pLCMV-puro plasmid (kindly provided by P.M. Chumakov) using the specific primers 5′-AAGGAAAAAAGCGGCCGCTGTGAAGGAAGCCAACCA-3′ (NotI endonuclease site is underlined) and 5′-AAAACTGCAGTTCCGGCTCGTATGTTGTGT-3′ (PstI endonuclease site is underlined). The resulting fragment was treated with the restriction endonucleases PstI and NotI and ligated to pNanoLuc-mSOG plasmid pretreated with the same restriction enzymes.

**Transfection of SK-BR-3 cells**

For transfections, plasmid DNA isolated from bacterial cells with the PureLinkTM kit (Invitrogen) according to the manufacturer’s instructions was used. Transfection was performed using FuGENE® HD (Promega) according to the manufacturer’s recommendations (http://www.promega.com/techserv/tools/FugeneHdTool/). A day before the transfection, the cells were seeded at a density of 105 cells/ml in a complete growth medium without antibiotics. FuGENE® HD and DNA were used in a 3: 1 ratio, and the concentration of the plasmid DNA during the formation of the complexes was 0.02 μg/μl. The volume of the medium that was added to the cells and contained FuGENE® HD–DNA complexes was 1/20 of the total volume of the growth medium. The complexes were prepared in a medium without serum and antibiotics, cultured at room temperature for 5–10 min and added to the cells. In the case of plasmids containing *miniSOG*, riboflavin (FMN cofactor) was added to the cells 6 h after the transfection. The optimal transfection conditions were determined in preliminary experiments by evaluating miniSOG fluorescence 24–48 h after the transfection using a fluorescence microscope.

**Sorting of transfected cells**

Cells expressing NanoLuc-miniSOG were collected 48 h after transfection using a BD FacsVantage sorter (BD, USA). For the sorting, the area of bright fluorescent cells was selected on a FL1-FL2 diagram so that it did not capture the cells that were fluorescent due to the presence of FMN in the medium (background FMN fluorescence). The sorted cells were seeded at a density of 105 cells/ml per well of a 96-well plate in 100 μl of a complete growth medium containing penicillin (50 U/ ml), streptomycin (50 μg/ml), kanamycin (100 μg/ml), and gentamicin (10 μg/ml) (all antibiotics are produced by “PanEco” Russia).

**Preparation of stable cell lines**

The concentration of puromycin (Sigma, USA) that caused the death of 100% of the cells in 14 days (0.25 mg/ml for SK-BR-3 cells) was detected during preliminary experiments. The medium in the plates with cultured cells was replaced with a fresh medium containing FMN and puromycin 48 h after transfection with the pNanoLuc-miniSOG-puro plasmid. Clones of the stably transfected cells were formed by day 14–15, after which the cells were passaged in the presence of puromycin for 3 months.

**Detection of NanoLuc luciferase luminescence**

The luminescence of NanoLuc luciferase and the NanoLuc-miniSOG fusion protein was evaluated 48–72 h after transfection on an Infinite M1000 Pro device (Tecan, Switzerland). Measurements were carried out using living cells in a complete RPMI medium without phenol red in 96-well plates with black walls (three repeats for each sample). The luciferase substrate furimazine (Promega) was added at concentrations of 30, 43, and 75 μM in the injection mode on an Infinite M1000 Pro device (Tecan, Switzerland). The delay after injection until the start of the analysis was 10 sec. Luminescence spectra were obtained for each experimental point in the wavelength range from 400 nm to 600 nm with a 4-nm increment and detection time of 100 msec. The obtained data were processed using the OpenOffice software, version 4.1.2. Mathematical data processing (smoothing with cubic splines) was used for the spectra plotting.

**Evaluation of the cytotoxic effect of NanoLuc-miniSOG in vitro**

The cytotoxicity of NanoLuc-miniSOG in the presence of furimazine was evaluated using the MTT test [25]. SK-BR-3 cells stably expressing the *NanoLuc-miniSOG *gene were seeded in a 96-well plate in the amount of 105 cells/ml of the medium in a volume of 200 μl of suspension per cell and cultured overnight. Then, the cells were supplemented with furimazine and incubated for 48 h. The medium was removed, 100 μl of a 3-[4,5-Dimethylthiazol-2-yl]-2,5-diphenyltetrazolium bromide (MTT, “PanEco”) solution in a McCoy’s 5A medium was added per well (0.5 mg/ml), and then the cells were incubated at 37°C and 5% CO_2_ for 1 h. Next, the MTT solution was removed, 100 μl of DMSO was added to the wells, and the plate was incubated on a shaker until complete dissolution of the formazan crystals. The optical absorption of the content of each well was measured on a tablet spectrophotometer Infinite M1000 (Tecan, Switzerland) at two wavelengths: 570 (experimental) and 650 nm (reference). The experiments were conducted in triplicate. Cell survival after incubation with furimazine was assessed based on the amount of formazan formed as a result of the reduction of the MTT solution by the cells and dissolved in dimethylsulfoxide (the amount of formazan corresponds to the number of living cells). The data were processed using the OpenOffice software, version 4.1.2.

## RESULTS AND DISCUSSION

For effective direct energy transfer from the oxidized form of the substrate to an acceptor (Förster resonance energy transfer), a number of conditions are required. First of all, an emission spectrum of the donor had to coincide as much as possible with the excitation spectrum of the acceptor. Secondly, the donor and acceptor had to be separated from each other by a distance not exceeding 10 nm [26].

Having performed an analysis of the published data, we found that the reaction of furimazine oxidation by NanoLuc luciferase of the deepwater shrimp *Oplophorus gracilirostris *results in an emission of light in the visible spectrum with an emission maximum at 460 nm [27]. The absorption maximum of the phototoxic flavoprotein miniSOG is 448 nm [24]. Thus, the oxidized form of furimazine (furimamide) and miniSOG offer a good donor-acceptor pair for bioluminescence resonance energy transfer. Superposition of the furimamide emission and miniSOG excitation spectra is shown in *Fig. 1C*.

In order to bring together the donor and acceptor spatially, we obtained a construct containing NanoLuc luciferase and miniSOG phototoxin genes connected by a short linker of 15 nucleotides within the same single reading frame under the control of the constitutive CMV promoter (*Fig. 1B*).

**Fig. 2:**
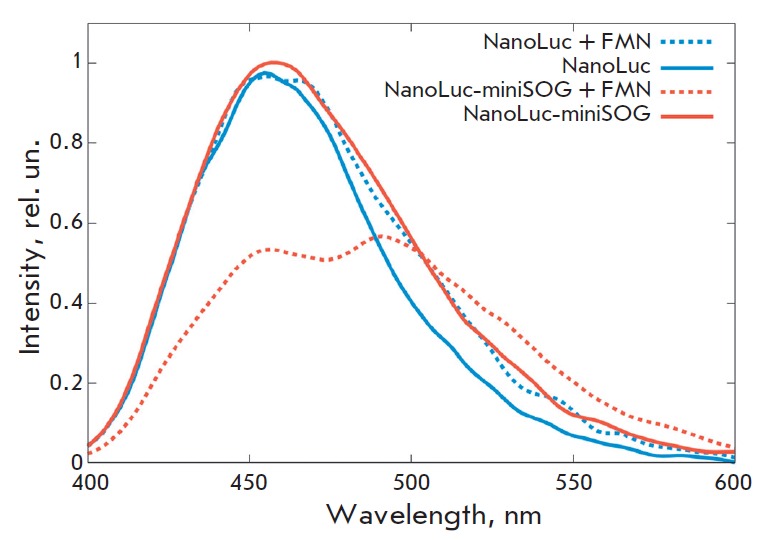
Emission spectra of the bioluminescence systems NanoLuc-furimazine (NanoLuc) and NanoLuc-furimazine-miniSOG (NanoLuc-miniSOG) in the presence of FMN.

The efficiency of this system was evaluated *in vitro *using a SK-BR-3 line transfected with the obtained construct. An analysis of the emission spectra of the transfected cells in the presence of furimazine demonstrated a peak at 460 nm, corresponding to the emission maximum of the oxidized form of furimazine *(Fig. 2)*.

FMN is known to be a cofactor of all phototropins (including flavoprotein miniSOG). The phototoxicity of miniSOG directly depends on its saturation with the cofactor: FMN enters an excited state under the impact of a blue light quantum, and all the energy of the excited state of FMN transfers into the generation of reactive oxygen species [24]. Therefore, riboflavin, which penetrates through the cellular membrane and turns into FMN as a result of phosphorylation by riboflavin kinase, was added at a concentration of 150 μM to the cells transfected with the pNanoLuc-miniSOG plasmid. Addition of riboflavin to the cells transfected with pNanoLuc-miniSOG in the presence of furimazine led to a decreased intensity of the peak at 460 nm and the appearance of a peak at 500 nm (miniSOG emission maximum), which indicates energy transfer from furimamide to miniSOG. We should note that addition of FMN to cells transfected with the plasmid containing the NanoLuc luciferase gene (without miniSOG) does not lead to the appearance of a 500 nm peak (*Fig. 2*).

In order to evaluate the cytotoxic effect caused by the NanoLuc–furimazine–miniSOG system, SK-BR-3 cells transfected with the pNanoLuc-miniSOG plasmid were sorted using a BD FacsVantage sorter (BD) 48 h after transfection. The selected cells were seeded in a 96-well plate for the assessment of the NanoLuc-miniSOG construct cytotoxicity in the presence of furimazine. However, the cells that had passed through the sorter and were exposed to the laser with a wavelength of 473 nm turned out to be not viable. We believe that short-time exposure to blue light (cell passage through the laser beam) was enough for miniSOG excitation and manifestation of its photoinduced cytotoxicity.

In order to circumvent this problem, we obtained a SK-BR-3 cell line stably expressing the NanoLuc-miniSOG construct. The selection of transfectants was carried out in the presence of the puromycin antibiotic as described in the “Experimental section.”

**Fig. 3:**
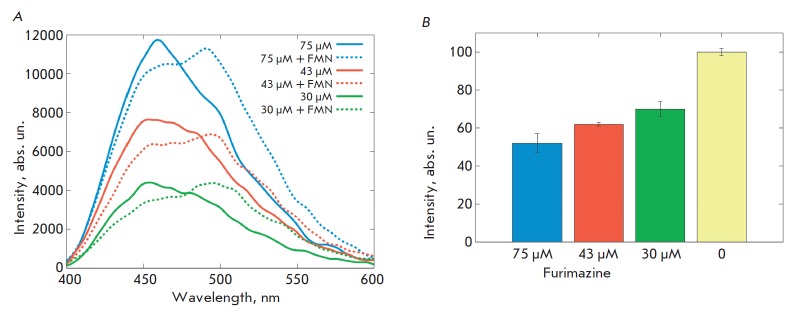
Functional characteristics of the bioluminescence system NanoLuc-furimazine-miniSOG. *A *– Dependence of NanoLuc-miniSOG emission spectra on furimazine and FMN concentrations. *B *– Dependence of the cytotoxic effect of the NanoLuc-miniSOG fusion protein on the furimazine concentration.

The analysis of the emission spectra of the cells containing the NanoLuc-miniSOG fusion protein in the presence of various concentrations of furimazine showed a peak at 460 nm, the intensity of which correlated with the substrate concentration (*Fig. 3A*). Addition of FMN to the cells led to the appearance of a peak at 500 nm, typical of a miniSOG emission maximum (*Fig. 3A*).

For a study of the cytotoxic effect of the “NanoLuc luciferase-furimazine-miniSOG phototoxin” system, SK-BR-3 cells stably expressing a NanoLuc-miniSOG hybrid construct were seeded in a 96-well plate and grown in the presence of FMN for 24 h. Further, furimazine was added to the cells at various concentrations and the cells were incubated at 37°C in a CO_2_ atmosphere for 48 h. The cytotoxic effect at a maximum concentration of furimazine was 48% (*Fig. 3B*).

It is known that miniSOG localized in mitochondria or in the plasma membrane causes the death of almost ; 100% of HelaKyoto cells under exposure to blue light (55 mW/cm2) [28]. Moreover, the unsaturated fatty acids contained in the plasma membrane in high amounts are the primary target for reactive oxygen species [29]. An additional factor contributing to the photo-induced damage to lipids is molecular oxygen, which is soluble in lipids. Thus, the photosensitizer is more likely to meet with molecular oxygen and generate ROS in a lipid environment than in water.

Taking into account the data in the papers [28, 29], we believe that the cytotoxic effect we identified in the course of our study can possibly be enhanced by using NanoLuc-miniSOG hybrid constructs with signals of various intracellular localization (mitochondrial, membrane, lysosomal). The systems based on a BRET-mediated activation of the photosensitizer will significantly enhance the capabilities of PDT by overcoming the problem of the “optical window” of biological tissues.

We have proved that cytotoxic flavoprotein miniSOG excitation by light emitted by the oxidized form of the luciferase substrate is possible, and shown that this system can be used for photo-induced cell death.

## CONCLUSION

This paper shows for the first time that it is possible to use bioluminescence resonance energy transfer to excite a genetically encoded photosensitizer. The light emitted by the oxidized form of the luciferase substrate renders the phototoxic protein miniSOG, which is part of the fusion with luciferase, into the excited state necessary for the generation of reactive oxygen species and cell death induction. The use of bioluminescence as an intracellular source of photosensitizer excitation in a cancer cell may become a solution to the problems of light delivery into deep regions of tissues and enhance the capabilities of photodynamic therapy of deep tissue tumors and metastasis.

## Abbreviations

ROS: reactive oxygen species
PCR: polymerase chain reaction
PDT: photodynamic therapy
FMN: flavin mononucleotide
BRET: bioluminescence resonance energy transfer
